# Relationship between latent trigger points, lower limb asymmetry and muscle fatigue in elite short-track athletes

**DOI:** 10.1186/s13102-023-00719-y

**Published:** 2023-09-12

**Authors:** Mariusz Konieczny, Elżbieta Skorupska, Przemysław Domaszewski, Paweł Pakosz, Marta Skulska, Pablo Herrero

**Affiliations:** 1 Faculty of Physical Education and Physiotherapy, Opole University of Technology, Prószkowska 76, Opole, 45-068 Poland; 2 Department of Physiotherapy, Poznan University of Medical Sciences, Poznan, 61-701 Poland; 3 Department of Health Sciences, Institute of Health Sciences, University of Opole, Katowicka 68, Opole, 45-060 Poland; 4 Faculty of Health Sciences, IIS Aragon, University of Zaragoza, Domingo Miral, s/n, Zaragoza, 50009 Spain

**Keywords:** Myofascial pain, Electromyography, Ground reaction force, Elite athletes, Body balance

## Abstract

**Background:**

Short-track speed skating movement involves asymmetric overloading of the lower left side of the body. The gluteus maximus fatigue limits the physical and mental athletic capacity to perform set tasks. A possible link between the presence of latent trigger points (LTrPs) and muscle fatigue development/persistence has been posited. The aim of the study was to determine whether elite short-track speed skating can result in the impairment of the musculoskeletal system of the lower limbs.

**Methods:**

Elite short-track athletes as the experimental group (EXP) = 9, 19.5 ± 1.8 years, and healthy subjects as the control group (CON) = 18, 20.8 ± 1.2 years, were tested for: (i) lower limb loading asymmetry using ground reaction force (GRF) measurements during quiet standing, (ii) gluteus maximus fatigue measured with surface electromyography (sEMG) during the Biering-Sorensen test, and (iii) LTrPs presence in the 14 examined muscles of the pelvic girdle and lower limbs.

**Results:**

There were between-group differences in the number of LTrPs, with the EXP group (left lower limb (LLL) n = 18, right lower limb (RLL) n = 9) showing more LTrPs compared to the CON group (LLL n = 2, RLL n = 1), (p < 0.001), and within-group differences in the EXP group only (p < 0.001). There were also significant differences in muscle fatigue for the left side (p < 0.001) both between the groups and within the EXP group (p ≤ 0.001). The vertical ground reaction force (GRF) measurement showed a loading rate of 2% (p = 0.013) in the athletes’ LLL exclusively.

**Conclusions:**

The study confirmed an increased prevalence of LTrPs, increased muscle fatigue and left-sided limb load asymmetry in elite short-track athletes.

**Trial registration:**

The study was conducted in accordance with the Declaration of Helsinki and approved by the Ethics Committee of the Poznan University of Medical Sciences (Resolution No 110/22 of 10 March 2022). Trial registration: 20/07/2022, Trial Id: ACTRN12622001016729.

## Background

Short-track speed skating is an Olympic sport characterized by a specific athlete’s position and counterclockwise high-speed movement on an icy track. It has been posited that it can lead to asymmetric muscle demands and subsequent overload and fatigue in the lower limb muscles. Moreover, the pattern of changes observed in the lower limbs has been proved to be asymmetric, with the left side affected more frequently, which is revealed by, e.g. impaired muscle oxygenation or changed muscle activation [1, 2]. Additionally, the fatigue of the gluteus maximus (GM) muscle has been confirmed as a common problem in this sport discipline [3, 4]. The gluteus maximus muscle plays an important role in maintaining the proper position of a short-track skater, acting as a hip extensor along with the hamstring muscles. Because of it, the fatigue and/or weakness of the GM muscle, especially at an elite level, can be associated with different injuries such as anterior cruciate ligament injuries or hamstring strains [5]. However, studies on fatigue and weakness in other muscles involved during short-track speed skating are limited. Some authors hypothesize that the increased number of latent trigger points (LTrPs) is one of the reasons for muscle fatigue and muscle weakening.

Trigger points (TrPs) are defined as hyperirritable painful spots located within a taut band of skeletal fibers. Although two types of TrPs have been distinguished depending on whether they provoke spontaneous pain (active) or not (latent), it is known that both latent and active TrPs can be responsible for muscle inhibition, weakness and fatigue. The pathomechanism of TrPs is not yet fully understood. There is an ongoing debate about the nature of pain pathomechanism, which has been argued to be either nociplastic or nociceptive [6, 7]. Previously, symptoms provoked by TrPs were categorised as functional pain. Currently, functional pain is referred to as “nociplastic pain” [6, 8].

In 2016, the International Olympic Committee pointed out nociplastic pain as a new opportunity for better diagnosis and pain prevention and a cause of potential injury in high-performance sports [9]. Despite the ongoing debate about TrPs pathomechanism, the clinical meaning of TrPs continues to be a relevent issue, particularly for elite athletes.

Research shows that muscle fatigue can affect kinematic changes in movement and can alter muscle activation patterns to maintain optimal performance levels. Taking into account the limited number of studies on the incidence of TrPs in short-track athletes and how TrPs occurrence may affect their pattern of movement, this study aimed to examine whether the incidence of LTrPs in short-track athletes was higher than in non-athlete controls and which were the main impairments associated with LTrPs presence.

## Methods

Nine elite athletes (n = 9), members of the Polish national short-track team, aged 19.5 ± 1.8 years, participated in the experimental group (EXP) study. The athletes had at least ten years of competitive experience and, at the time of the study, they had no injury and were participating in normal training. In the study, we diagnosed the presence of latent TrPs based on the Delphi recommendation [10]. The control group (CON) consisted of 18 healthy non-athletes, aged 20.8 ± 1.2 years and not training or having an injury. Fourteen muscles on each side of the body were subject to examination: gluteus maximus, gluteus medius, gluteus minimus, quadratus lumborum, adductor longus, adductor magnus, tensor fasciae latae, vastus lateralis, vastus medialis, gracilis, rectus femoris, biceps femoris, semitendinosus and semimembranosus.

The ground reaction force (GRF) symmetry of the lower limbs during quiet standing was measured under the left and right foot using a force plate (9286AA; Kistler Group, Winterthur, Switzerland) at a sampling frequency of 100 Hz. Three measurements were taken and the mean value was calculated. The percentage of GRF on the left lower limb was calculated using the formula GRFLLL/(GRFLLL + GRFRLL) [11].

**Fig. 1:**
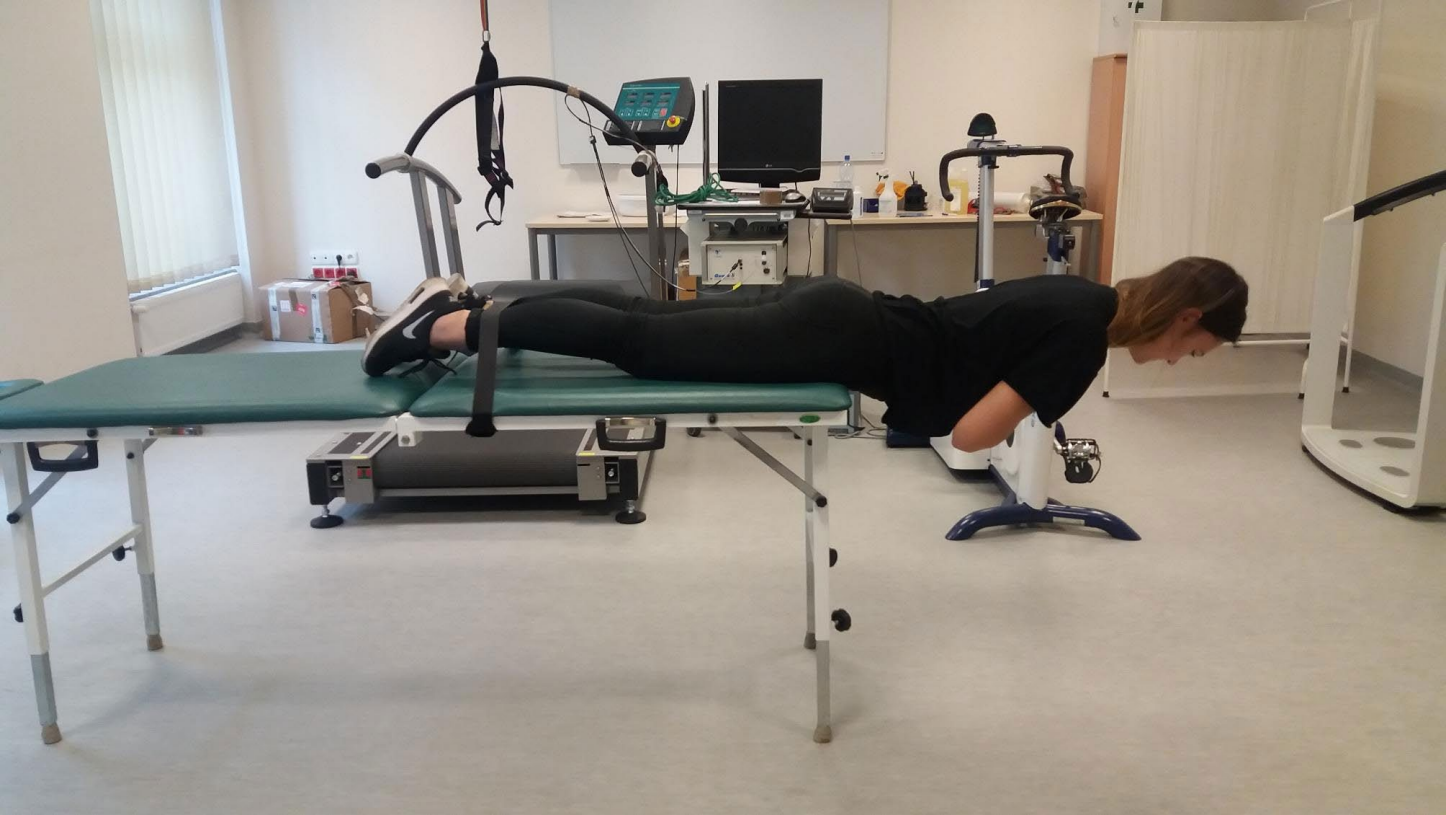
Body position in the Biering-Sorensen test

Muscle fatigue was measured using sEMG (slope frequency) of the GM activity according to the Biering-Sorensen test protocol that ensures isometric and symmetric muscle tension of the examined body segment due to the assumed body position (Fig. 1.). This test (Biering-Sorensen test) has been shown to be reliable [12, 13] and has been repeatedly used in GM muscle fatigue studies [14]. It is also a test in which simultaneous and symmetrical isolation of both gluteal muscles (GM) occurs [15, 16]. A 16-channel sEMG system (manufactured by NORAXON DTS) that recorded signals at a sampling rate of 1500 Hz was used. Signal processing was performed using NORAXON MR-XP 1.07 Master Editions software. The skin was cleaned to prepare the muscles for analysis and the electrodes (Ag/AgCl) were taped according to the SENIAM methodology [17]. Median frequency (MF) was calculated in 1s time windows using the power spectral density that was estimated by the Welch averaged periodogram method (PWELCH Matlab function). Slopes and intercepts were calculated using linear regression on the calculated MF values as a function of time.

Statistical analysis: The mean number of TrPs was measured using the Independent Samples Mann-Whitney U test and the slope frequency values measured by sEMG (muscle fatigue) were analyzed with Repeated Measures ANOVA. The two groups (EXP and CON) constituted the between-subject factor and the two sides (left lower limb and right lower limb) constituted the within-subject factor. In case that interaction was significant, Tukey’s post hoc tests were applied for pairwise comparisons of the four-factor combinations. An Independent Samples t-test was used to analyse the significance of differences in % ground reaction force (GRF[%]) of the left lower limb. Jamovi 2.0 was used for statistical analysis.

## Results

The short-track athletes group (EXP) presented a higher number of LTrPs in the left lower limb (n = 18) compared to the opposite right lower limb (n = 9), whereas in the non-athletes group (CON) there were only three LTrPs. Apart from the difference in the number of LTrPs, the EXP group showed significant differences in the distribution of LTrPs between the two sides (U = 14, p = 0.012, r = 0.59). On the contrary, in the CON group, there were no significant differences between the two sides. Moreover, when differences between the groups were analyzed, there were significant differences between the EXP and CON groups: left lower limb (U = 2, p < 0.001, r = 0.89), right lower limb (U = 30.5, p < 0.001, r = 0.65) (Table 1).

**Table 1:** Asymmetric overloading of the left lower limb characterized short-track athletes

	Number of TrPs (n = 30)		p	p		Slope fatigue		p sides	p groups	GRF [%]	p groups
	left	right	left vs. right	left	right	left	right	left vs. right		left vs. right	
EXP n = 9	18	9	0.014	< 0.001	< 0.001	− 0.08	− 0.04	< 0.001	0.001	48	< 0.013
CON n = 18	2	1	0.57			− 0.02	− 0.03	0.908		50	

EXP – experimental group (short-track elite athletes); CON – control group (healthy non-athletes); LTrPs – latent trigger points; GRF[%] – ground reaction force contribution of the left lower limb was calculated according to the formula GRFLLL/(GRFLLL + GRFRLL); the p value < 0.05- of the differences between the right lower limb vs. left lower limb. Slope frequency – muscle fatigue measured for the gluteus maximus muscles during the Biering-Sorensen test

**Fig. 2:**
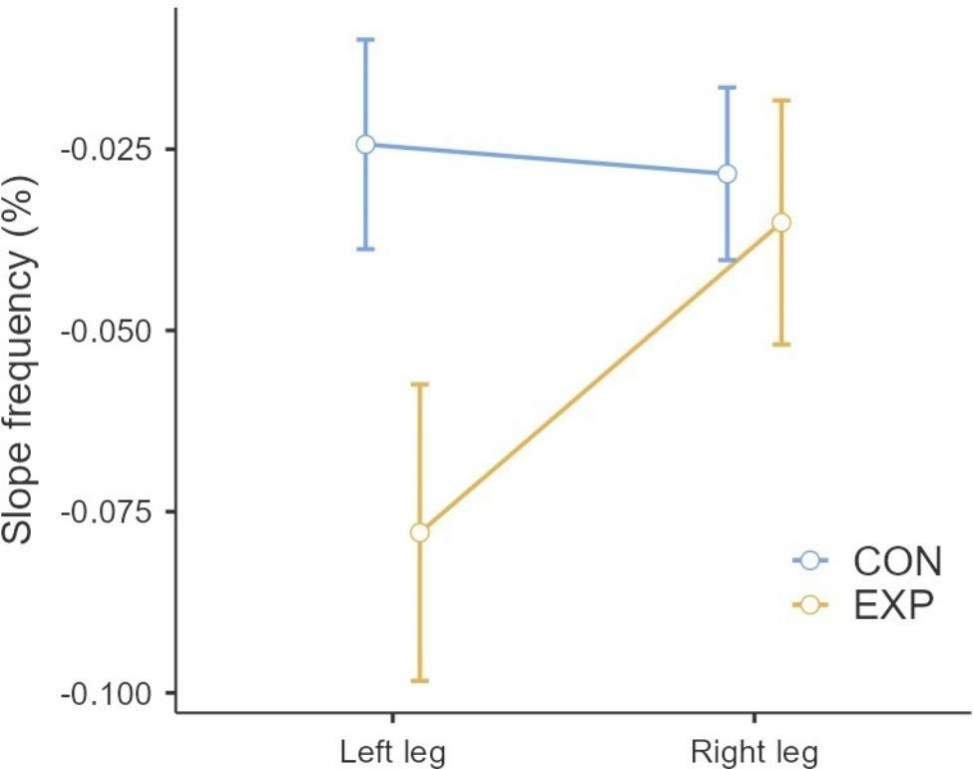
Graphical analysis of the lower limb asymmetric overloading

Regarding muscle fatigue, there were statistically significant differences between groups (p < 0.001; F = 9.41; η²p = 0.274), as well as within-group differences for the EXP group in the left GM (p < 0.001, t = − 4.99). Figure 2 shows a graphical analysis of the mean and confidence interval measured for the percentage difference of the slope frequency (sEMG) of the GM muscles’ bioelectrical activity examined during the Biering-Sorensen test. A greater negative value of the percentage difference in slope frequency indicates a greater decrease in frequency, which is interpreted as an increase in muscle fatigue. The graph shows this dependency for the EXP group.

The measurement of ground reaction forces during quiet standing (GRF[%]) of the short-track athletes (EXP) showed a significant difference in the weight shift to the right lower limb (p = 0.013; t = 2.95), which contrasts with the symmetry between the lower limbs observed for the CON group.

## Discussion

This is the first study that demonstrates specific musculoskeletal changes in the left lower limb of short-track athletes compared to healthy subjects. The athletes presented a higher number of LTrPs in the pelvic girdle and lower limb muscles, with a significantly higher number for the left side of the body compared to the control group. This was coincident with a fixed right shift of the quiet standing position and the fatigue of the GM muscle, which could be associated with the skating movement dynamics on the ice. An increased number of LTrPs confirmed for the short-track athletes is in line with the results published by Gomez et al. [18], who found that athletes tend to have a higher number of latent TrPs in the lower limbs compared to sedentary individuals. The results also support Ge and Arendt-Nielsen’s findings [19], according to which long-term or low-load repetitive muscle activity or prolonged ischemia can damage myofibrils and promote latent TrPs formation. Related to this, it has been proved that the presence of TrPs is associated with altered kinematics, muscle activation and muscle fatigue [20–22], which may help to explain why an increased number of LTrPs could be associated with other phenomena found in our study such as muscle asymmetry and muscle fatigue. To date, only a few studies have investigated the incidence of latent TrPs among athletes. Thus, it is difficult to compare our study results. However, it seems that the meaning of TrPs is relevant, not only for muscle fatigue but also for pain, which was confirmed among elite swimmers [23].

In our study, athletes also exhibited greater GRF on the right lower limb compared to the control group, showing an asymmetry which could be explained by LTrPs existence in the pelvic girdle and lower limb muscles. There are no previous studies comparing muscle symmetry or the GRF applied during the quiet standing position for short-track athletes and controls. Our results differ from the normative data in asymptomatic individuals [24] that show symmetry between the lower limbs in the quiet standing position. Moreover, our findings contrast with a study carried out on athletically inactive subjects by Gutnik [25], who found no differences in loading symmetry when compared with a healthy group of individuals. The only study with similar results was the research performed by Gade et al. [26], who found asymmetry in the lower limbs loading in one out of three healthy subjects but the results were based on a small study group (n = 9) and should be interpreted with caution.

A possible explanation of the observed asymmetry is the specific athlete’s position and counterclockwise high-speed skating movement on an icy track. As confirmed in the present study, lower limb asymmetry in short-track athletes is an undesirable effect that may result in an increased risk of injury due to limited athletes’ movement strategies [27, 28]. Moreover, the shift of GRF to the right lower limb found in our study can be related to the increased number of LTrPs in the left pelvic girdle and lower limb muscles, similarly to what was found by Jones et al. [29], who stated that the increased number of TrPs in the lower limbs resulted in impaired postural control. Other authors hypothesized that the shift to the non-fatigued lower limb, confirmed by quiet two lower limbs measurement, can reflect adaptive changes of the body position due to muscle fatigue [30], which could also be related to an increased LTrPs prevalence in the left side.

According to the literature, the presence of LTrPs can provoke the development of muscle fatigue four times faster than in non-TrP muscles, probably due to the simultaneous overloading of active motor units in the vicinity of TrPs [22]. Additionally, the relationship between muscle fatigue and latent TrPs has been observed during push-up activities for the upper trapezius muscle in 80% of 64 healthy participants (with LTrPs in their upper trapezius muscles [31].

Finally, our results support the claim that the movement characteristic of short-track skating on ice (counterclockwise) predisposes athletes to the overload of the left side and consequent muscle fatigue and LTrPs development. This can further lead to an increased risk of lower limb injury and/or functional pain development due to impaired body control and muscle alterations. Furthermore, it has been proved that other muscles can develop fatigue due to asymmetrical movements in some sports disciplines [30, 32, 33] and that unilateral foot muscle fatigue results in asymmetry in the quiet body position [30]. All this is important for the development of sports medicine, injury prevention and advanced athlete management systems. Importantly, the management of undiagnosed functional pain, which is ultimately redefined as nociplastic pain, is a new idea for elite sports management established by the International Olympic Committee [7]. The concept of TrPs as a reason for nociplastic pain related to muscle is under development but it seems to be a promising biomarker of nociplastic pain occurrence among athletes [34].

## Conclusions

The study confirmed an increased incidence of LTrPs in elite short-track athletes and an asymmetric LTrPs distribution in the lower limbs, with the left side affected more frequently. All athletes exhibited an asymmetric decrease in slope frequency in the left gluteal muscle, which suggests greater fatigue and an asymmetric distribution of body load compared to the control group.

## Data Availability

The datasets generated and/or analysed during the current study are not publicly available. However, the data are available from the corresponding author on reasonable request.

## List of abbreviations

LTrPs: Latent trigger points
GRF: Ground reaction force
TrPs: Trigger points
sEMG: Surface electromyography
GM: Gluteus maximus muscle
MF: Median frequency
